# Atypical adenomatous hyperplasia (adenosis) of the prostate: a case report with review of the literature

**DOI:** 10.1186/1746-1596-3-34

**Published:** 2008-08-12

**Authors:** Henry B Armah, Anil V Parwani

**Affiliations:** 1 Department of Pathology, University of Pittsburgh Medical Center, Pittsburgh, PA, 15213, USA

## Abstract

A 62-year-old male presented with symptoms of urinary obstruction and elevated serum prostate-specific antigen level of 3.61 ng/mL. Prostate needle biopsies showed benign prostatic tissue with a focus of crowded glands with minimal cytological atypia, fairly well-circumscribed with infiltrative appearance of glands at the edges. This focus had both small and larger glands with similar histological features. This focus was strongly positive for alpha-methylacyl-coenzyme A-racemase (AMACR), but showed scattered patchy staining with basal cell markers (p63 and CK903/34βE12). Hence, the final histologic diagnosis was benign prostatic tissue with a focus of florid adenosis. Two subsequent follow-up prostate needle biopsies performed six and 12 months later both showed benign prostatic tissue with atrophic changes. This case highlights the utility of these three immunostains (AMACR, p63 and CK903/34βE12) in the accurate diagnosis of adenosis of the prostate on needle biopsy, and avoiding its misinterpretation as prostate adenocarcinoma.

## Background

Atypical adenomatous hyperplasia (AAH) or adenosis of the prostate is a pseudoneoplastic lesion that can mimic prostate adenocarcinoma because of its cytologic and architectural features [1-3]. For many years, atypical epithelial lesions of the prostate have been known to occur, but much refining of this knowledge has evolved over the last two decades. Initially two lesions, prostatic intraepithelial neoplasia (PIN) and AAH, were assumed to be precursors of prostatic adenocarcinoma [1-3]. However, PIN now remains as the only well-proven preneoplastic condition with clinical significance. AAH is no longer considered a premalignant lesion but rather a benign small glandular process of the transition zone that simulates acinar adenocarcinoma [1-3]. Since AAH occurs predominantly in the transition zone, which is only rarely sampled in needle biopsy, it is uncommon to see examples of this lesion in biopsy specimens. However, as the sampling of the transition zone of the prostate has become more frequent recently with ultrasound-guided multiple segmental prostate biopsies [4] the practicing surgical pathologists must be aware of the histologic features of AAH of the prostate in needle biopsy specimens, in order to avoid misinterpretation of AAH of the prostate, a benign lesion, as prostate adenocarcinoma. Additionally, because some patients showing raised prostate specific antigen levels and negative peripheral zone biopsy may present with transition zone prostatic adenocarcinoma, sampling of the transition zone of the prostate by needle biopsy and the identification of AAH are likely to increase [4].

## Case presentation

A 62-year-old male was referred to his urologist for symptoms of urinary obstruction and an elevated serum prostate-specific antigen (PSA) level of 3.61 ng/mL. Digital rectal examination and ultrasound revealed an ill-defined nodule in the prostate suggestive of malignancy. A needle biopsy was performed.

**Figure 1:**
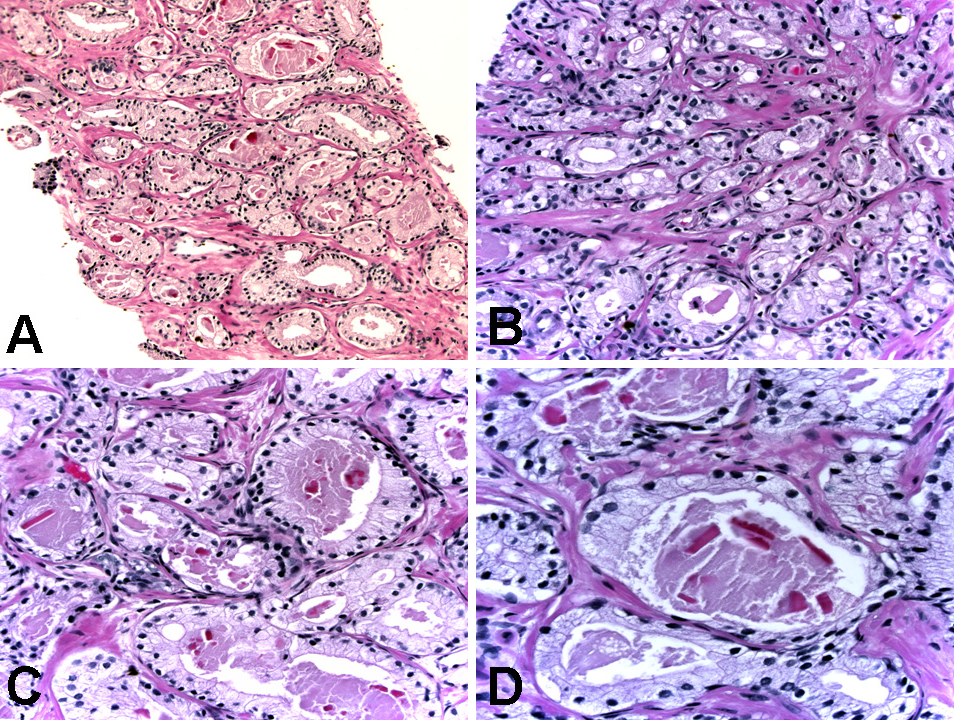
**Histologic (hematoxylin-eosin) findings of atypical adenomatous hyperplasia of the prostate**. (A) Crowded haphazardly arranged variably sized glands with infiltrative appearance of glands at the edges. Original magnification × 200. (B) Predominantly small glands lined by epithelial secretory cells with clear eosinophilic cytoplasm and minimal cytological atypia. Original magnification × 400. (C) Predominantly large glands lined by epithelial secretory cells with clear eosinophilic cytoplasm and minimal cytological atypia. Original magnification × 400. (D) Predominantly large glands lined by epithelial secretory cells with clear eosinophilic cytoplasm, minimal cytological atypia with inconspicuous nucleoli, few focally prominent basally located cells with dense amphophilic cytoplasm, and luminal eosinophilic crystalloids. Original magnification × 600.

The histologic section of one needle biopsy was characterized at low-power examination by the replacement of normal prostatic tissue by a proliferation of haphazardly arranged glands, partially arranged in an ill-defined nodule. The lesions had an infiltrative aspect at their edge, but the glands were not admixed with normal prostatic acini. At medium power, the proliferating glands were often slit-like, variably sized and shaped, alternating small and rounded acini with elongated and branching ones (Figure 1A &1B). Occasional solid nests and cords could be seen. The atypical acini were lined by epithelial secretory cells with clear eosinophilic cytoplasm (Figure 1C). The nuclei were regular, rounded to oval, and slightly larger than those of the adjacent normal prostatic acini. There were inconspicuous nucleoli. There were a few focally prominent basally located cells with dense amphophilic cytoplasm. Eosinophilic crystalloids were present within some acinar lumina (Figure 1C &1D).

**Figure 2:**
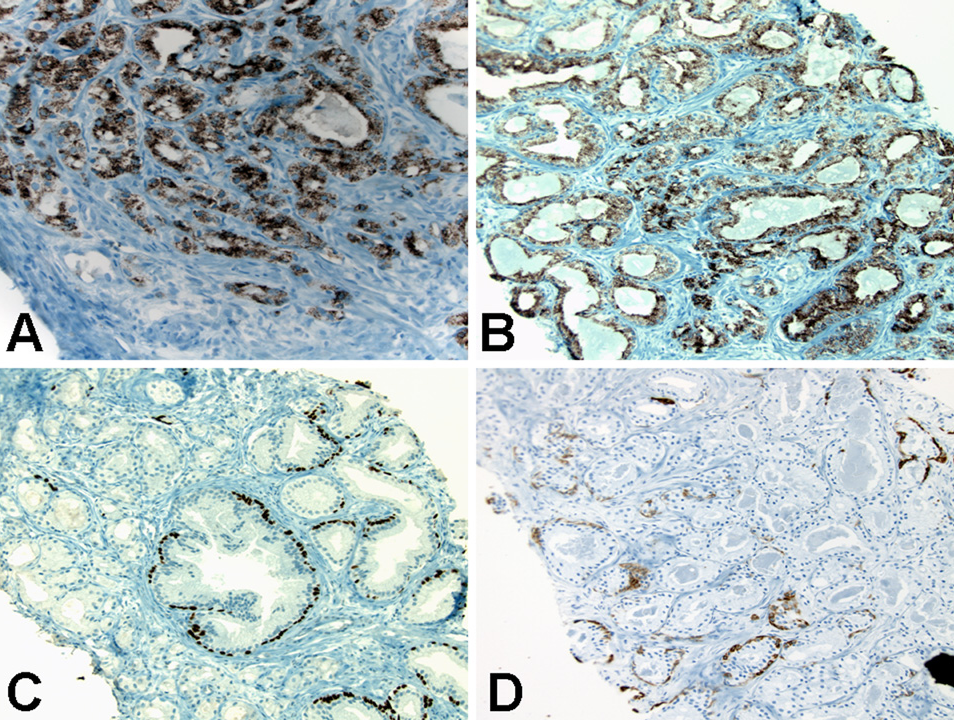
**Immunohistochemical findings of atypical adenomatous hyperplasia of the prostate**. (A) Strong reactivity for alpha-methylacyl-coenzyme A-racemase in predominantly small glands. Original magnification × 200. (B) Strong reactivity for alpha-methylacyl-coenzyme A-racemase in predominantly large glands. Original magnification × 200. (C) Patchy reactivity for p63 in both small and large glands. Original magnification × 200. (D) Patchy reactivity for high-molecular-weight cytokeratin CK903/34βE12 in both small and large glands. Original magnification × 200.

Immunohistochemical (IHC) stains revealed strong reactivity for alpha-methylacyl-coenzyme A-racemase (AMACR) in both small and large glands (Figure 2A &2B), patchy reactivity for p63 (Figure 2C) and patchy reactivity for high-molecular-weight cytokeratin CK903/34βE12 (Figure 2D).

The final histologic diagnosis was benign prostatic tissue with a focus of florid AAH. Two subsequent follow-up prostate needle biopsies performed six and 12 months later both showed benign prostatic tissue with atrophic changes.

## Discussion

AAH of the prostate is a microglandular lesion and a recognized mimicker of small acinar adenocarcinoma [1-3]. AAH is characterized by a proliferation of prostatic glands with abnormal architectural patterns, but without significant cytologic atypia. AAH of the prostate is usually an incidental finding in transurethral resections or simple prostatectomies performed in the clinical setting of benign prostatic hyperplasia. The prevalence of AAH in transurethral prostatectomy specimens without cancer ranges from 1.6% to 7.3% [5] compared to its prevalence of 0.8% in needle biopsy specimens [6]. Their rarity in needle biopsies of the prostate is attributable to the fact that sampling of the transition zone is not common in needle biopsies. Although, AAH can be diagnosed throughout the prostate, it is most often located in the transition zone of the prostate in intimate association with benign nodular hyperplasia [7]. Since the frequency of needle biopsies, including tissue from the transition zone, is likely to increase due to the introduction of ultrasound-guided multiple segmental prostate biopsy [4], knowledge of the main diagnostic histologic features of AAH would represent an important issue in genitourinary surgical pathology. The diagnosis of AAH in needle biopsies, as seen in the case herein presented, relies on both histologic features in hematoxylin and eosin-stained slides and immunohistochemical features.

Microscopically, AAH is a localized proliferative lesion consisting of small amounts of atypical epithelial cells arranged in irregular glandular patterns, often within or adjacent to typical hyperplastic nodules [8]. At low magnification, it is usually partially circumscribed with a pushing rather than infiltrating border, although the small acini may show a limited degree of infiltrative features at the margins. The individual glands are closely packed but separate and show no evidence of fusion. The glands show some variation in size and shape and are lined by cuboidal to low columnar cells with moderate to abundant clear or lightly eosinophilic cytoplasm [6,9]. The basal cells are usually recognized at least focally. The luminal borders are often irregular and somewhat serrated in contrast to the rigid borders that typify small acinar carcinoma. The lumens are often empty but may contain corpora amylacea and in some instances luminal eosinophilic crystalloids [5,9]. The nuclei are round to oval, slightly enlarged, and with uniform fine chromatin and inconspicuous or small nucleoli [9]. AAH can be difficult to distinguish from low-grade prostatic adenocarcinoma (Gleason pattern 1 or 2) because both are located in the transition zone and show small acinar proliferation and intraluminal crystalloids [9]. The two distinguishing features of AAH are the lack of significant cytological atypia and the presence of patchy basal cells, which can be demonstrated by patchy immunostaining for high molecular-weight cytokeratin (CK903/34βE12) or p63 [7-9]. In contrast, prostatic adenocarcinoma usually shows notable nuclear atypia, lacks basal cells, and rarely expresses high molecular weight cytokeratin [10]. Yang and colleagues [11] found that AMACR was focally expressed in 10% of cases and diffusely positive in only 7.5% of cases of AAH. The biological significance of AMACR expression in a small subset of AAH remains to be determined. AAH differs from sclerosing adenosis, another benign mimicker of prostate adenocarcinoma, since sclerosing adenosis displays myoepithelial features of the basal cells and an exuberant stroma of fibroblasts and loose ground substance [12].

While circumstantial evidence exists, there is lack of proof of a relationship between AAH and adenocarcinoma. It has been suggested that AAH is a precursor of some low-grade transition zone carcinomas but the lack of an increased prevalence of AAH in prostate glands with transition zone carcinoma argues against this hypothesis. Clearly, there is less evidence linking AAH to carcinoma than there is for high-grade PIN and cancer. Therefore, the major importance of AAH is its potential for being misdiagnosed as adenocarcinoma. Biochemical and molecular analyses of AAH have generated inconclusive results. There is limited data that AAH has a proliferation rate higher than hyperplasia but lower than adenocarcinoma [1,7,8,13]. By the use of fluorescent *in situ *hybridization analysis, chromosomal anomalies were seen in only 9% of AAH cases, compared with 55% of prostatic adenocarcinoma cases [13]. Two independent studies showed that AAH contains genetic alterations commonly found in early prostatic carcinoma, with changes being reported in 47% or 12% of AAH cases, respectively [8,14]. Recent cytogenetic analyses have detected abnormalities of chromosome 8 in a very small proportion (4–7%) of AAH cases [7,8]. The recent finding of molecular alterations in AAH including immunoreactivity for AMACR, a marker linked to prostate adenocarcinoma, suggests that at least a subset of AAH cases might be related to prostate carcinoma of the transition zone [11,13,14].

The widespread use of PSA screening has led to an increase in prostate needle biopsies and, subsequently, an increase in earlier detection of prostate carcinoma. This trend has also led to an increase in the number of equivocal diagnoses on prostate biopsy specimens. Surgical pathologists must make critical decisions on an increasing number of prostate needle biopsy specimens with only small foci of atypical glands. In this setting, the mimics of prostate cancer must be distinguished from a small focus of adenocarcinoma. The distinction of benign small acinar proliferations (benign mimickers of cancer) from atypical acinar proliferations suspicious for cancer is crucial, since the subsequent clinical approach is different. Biopsies harboring a small focus of atypical glands frequently represent an under-sampled cancer and a subsequent biopsy will show cancer in up to 50% of cases [15]. In contrast, following a diagnosis of benign mimickers of cancer (such as atrophy or AAH), a re-biopsy is usually not indicated.

## Conclusion

AAH is a transition zone lesion of the prostate that can simulate small acinar carcinoma. By itself positive immunostaining for AMACR is not diagnostic for carcinoma because the latter is also positive in high-grade PIN and some benign mimickers of adenocarcinoma. The case herein presented highlights the utility of AMACR, p63 and CK903/34βE12 immunostaining in the accurate diagnosis of adenosis of the prostate, a benign mimicker of prostate adenocarcinoma.

## Competing interests

The authors declare that they have no competing interests.

## Authors' contributions

HBA participated in the histopathological evaluation, performed the literature review, acquired photomicrographs and drafted the manuscript. AVP conceived and designed the study, gave the final histopathological diagnosis and revised the manuscript for important intellectual content. Both authors read and approved the final manuscript.
